# Evaluating the Safety and Efficacy of Ticagrelor vs. Clopidogrel Following Percutaneous Coronary Intervention in Chronic Coronary Disease

**DOI:** 10.7759/cureus.77610

**Published:** 2025-01-18

**Authors:** Muhammad Abdur Rauf, Muhammad Ziyad, Nida Mahmood, Rafi Ullah, Farooq Ahmad, Ikram Ullah, Syed Muzammil Shah

**Affiliations:** 1 Cardiology, Kuwait Teaching Hospital, Peshawar, PAK; 2 Health, Khyber Medical University, Peshawar, PAK; 3 Cardiology, Khyber Teaching Hospital, Peshawar, PAK; 4 Cardiology, Lady Reading Hospital and Medical Teaching Institute, Peshawar, PAK

**Keywords:** chronic coronary disease, clopidogrel, mace, percutaneous coronary intervention, ticagrelor

## Abstract

Objective

This study aimed to compare the safety and efficacy of ticagrelor and clopidogrel in reducing major adverse cardiovascular events (MACE) among patients undergoing percutaneous coronary intervention (PCI) for chronic coronary disease. Additionally, secondary endpoints, including adverse events such as major bleeding, minor bleeding, and dyspnea, were assessed to evaluate the overall safety profile of both antiplatelet therapies.

Methodology

A prospective cohort study was conducted at Kuwait Teaching Hospital, Peshawar, Pakistan, enrolling 300 patients (150 receiving ticagrelor and 150 receiving clopidogrel) from July 2023 to June 2024. Patient selection was based on predefined inclusion and exclusion criteria, ensuring a homogeneous study population. Randomization was not applied, and treatment allocation was guided by physician discretion and clinical indications. Baseline characteristics, primary clinical outcomes (MACE), and secondary endpoints (major bleeding, minor bleeding, and dyspnea) were assessed. Statistical analysis was performed using chi-square tests for categorical variables and independent t-tests for continuous variables, with statistical significance set at p < 0.05.

Results

Ticagrelor significantly reduced the incidence of stent thrombosis compared to clopidogrel (8 (5.0%) vs. 20 (13.3%); p = 0.029, χ² = 4.78), indicating a 62.4% relative risk reduction and suggesting superior thrombotic protection in PCI patients. Although revascularization rates were lower with ticagrelor (10 (7.0%) vs. 18 (12.0%); p = 0.169, χ² = 1.89), the difference was not statistically significant, but the trend suggests a potential clinical benefit in reducing repeat interventions. Major bleeding was higher in ticagrelor users (15 (10.0%) vs. 9 (6.0%); p = 0.287, χ² = 1.13), aligning with its pharmacodynamic profile and underscoring the need for careful risk stratification in bleeding-prone patients. Dyspnea occurred more frequently with ticagrelor (12 (8.0%) vs. 5 (3.3%); p = 0.134, χ² = 2.25), likely due to adenosine-related effects, highlighting the importance of monitoring patients with respiratory conditions. Minor bleeding rates were comparable (21 (14.0%) vs. 15 (10.0%); p = 0.374, χ² = 0.79), indicating no significant difference in less severe bleeding events. Baseline characteristics, including age, BMI, smoking history, diabetes, and hypertension, were statistically similar (p > 0.05), ensuring comparability between the two groups and reinforcing that observed differences in clinical outcomes were treatment-related rather than due to baseline variability.

Conclusions

Ticagrelor demonstrated superior efficacy in reducing MACE, particularly stent thrombosis, but was associated with higher rates of bleeding and dyspnea. Individualized treatment strategies involve risk stratification, where high ischemic-risk patients benefit most from ticagrelor, while those prone to bleeding may require de-escalation to clopidogrel. Dyspnea and bleeding can impact adherence and quality of life, leading to premature discontinuation. Close monitoring, early symptom recognition, and shared decision-making are essential to optimize therapy, ensuring patients receive maximum benefit while minimizing adverse effects.

## Introduction

Over the past decade, significant progress has been made in the management of chronic coronary disease (CCD), particularly in optimizing percutaneous coronary intervention (PCI) outcomes and preventing thromboembolic complications through dual antiplatelet therapy (DAPT). Ticagrelor and clopidogrel are the primary antiplatelet agents used in DAPT, but their pharmacodynamic differences influence their clinical effectiveness. Ticagrelor, a direct-acting P2Y12 receptor antagonist, provides faster and more potent platelet inhibition compared to clopidogrel, which requires hepatic biotransformation for activation. These pharmacokinetic distinctions may contribute to ticagrelor’s superiority in reducing ischemic events. Their safety and efficacy in CCD patients post-PCI continue to be evaluated, particularly regarding bleeding risks and side effects in specific populations [1,2].

PCI is essential for revascularization in CCD and requires effective antiplatelet therapy. Ticagrelor has shown superior efficacy over clopidogrel in reducing major adverse cardiovascular events (MACE) in multiple trials, particularly among high-risk patients [3,4]. However, ticagrelor’s higher bleeding risk necessitates a careful risk-benefit analysis, especially in populations vulnerable to hemorrhagic complications.

Recent studies emphasize ticagrelor’s efficacy and safety over clopidogrel in East Asian populations, including Pakistan. While ticagrelor reduces ischemic events, it raises bleeding risks, highlighting the need for tailored guidelines [1,5]. Research from local settings such as Kuwait Teaching Hospital in Peshawar underscores the importance of optimizing these medications in Pakistan's healthcare system.

Evidence from Pakistan reveals the significance of evaluating ticagrelor’s safety. A study conducted in Peshawar demonstrated that while ticagrelor decreased thrombotic events post-PCI, it was associated with common side effects such as dyspnea and minor bleeding [6,7]. These findings stress the need for closely monitoring patients on ticagrelor, especially in resource-limited settings.

International studies have also provided valuable insights into these agents. A review by Maqbool et al. (2023) reported that ticagrelor reduced mortality and myocardial infarction rates compared to clopidogrel but increased the risk of major bleeding [8,9]. These results align with data from Pakistan, which indicates similar trends.

A prospective trial in East Asian populations explored the efficacy of low-dose ticagrelor to minimize bleeding risks while maintaining efficacy [10,11]. These strategies are particularly relevant in Pakistan, where balancing efficacy and safety is crucial due to diverse patient risk profiles.

Switching from ticagrelor to clopidogrel is feasible in patients with high bleeding risk. The TALOS-AMI trial supports this approach, demonstrating lower bleeding rates without compromising efficacy [12,13]. These findings suggest that personalized treatment plans may benefit certain patients post-PCI.

The ALPHEUS trial, which evaluated ticagrelor vs. clopidogrel in elective PCI patients, found no significant differences in periprocedural myocardial necrosis but reported increased minor bleeding with ticagrelor [14,15]. These results support the continued use of clopidogrel for low-risk elective PCI cases.

A systematic review by Dogan et al. (2003) assessed post-PCI monotherapy, concluding that ticagrelor improves net adverse clinical events compared to clopidogrel [16]. Another study highlighted ticagrelor’s advantages in ST-elevation myocardial infarction patients receiving thrombolysis, demonstrating enhanced thrombolysis in myocardial infarction flow and reduced ischemic complications [7].

This study aims to fill gaps in the literature regarding the population studied in Pakistan. Although international guidelines provide robust foundations for the use of ticagrelor and clopidogrel, their local applicability remains uncertain due to genomic, socioeconomic, and healthcare disparities. This study compares ticagrelor and clopidogrel in coronary artery disease patients undergoing PCI at Kuwait Teaching Hospital, Peshawar, Pakistan, providing evidence on clinical outcomes, safety profiles, and patient-reported adverse events to guide clinical decision-making in similar healthcare settings.

## Materials and methods

Study design and duration

This prospective cohort study was conducted at the Department of Cardiology, Kuwait Teaching Hospital, Peshawar, Pakistan over a one-year period (July 2023 to June 2024) to capture both short-term and mid-term clinical outcomes. Follow-up assessments were conducted at one and three months post-PCI, as these time points align with the peak incidence of MACE, bleeding events, and adherence-related issues. Patients were contacted through in-person visits, phone calls, and text message reminders to ensure follow-up adherence, with virtual consultations offered for those facing transportation barriers.

Sample size

Using OpenRCT, with 95% confidence, 80% power, and an expected outcome difference of 20% between groups, the sample size was calculated. A total of 300 patients were enrolled, with 150 in the ticagrelor group and 150 in the clopidogrel group.

Inclusion and exclusion criteria

Patients aged 18-75 years who underwent PCI for any form of CCD, including stable angina, prior myocardial infarction, and multivessel disease, and were prescribed either ticagrelor or clopidogrel as part of their DAPT were eligible.

Exclusion criteria included contraindications to ticagrelor or clopidogrel, a history of bleeding disorders, active malignancies, severe renal impairment (estimated glomerular filtration rate <30 mL/min/1.73m²), severe hepatic impairment (alanine transaminase/aspartate transaminase >3× upper limit of normal or clinical evidence of liver dysfunction), inability to provide informed consent, pregnancy, lactation, and participation in other clinical trials. Participation in other trials was verified through patient interviews and institutional medical record reviews to ensure accurate exclusion.

Data collection procedure

Hospital data were extracted from both electronic and paper records, including patient demographics, clinical history, procedural characteristics, and post-PCI outcomes. Follow-up data at one and three months included therapy adherence, clinical outcomes, and adverse events, collected through clinic visits and telephone interviews.

Definitions and assessment criteria

The primary outcome was the incidence of MACE, defined as a composite of cardiovascular mortality, myocardial infarction (diagnosed based on ECG changes, cardiac biomarkers, and clinical presentation), and stroke (confirmed through neurological assessment and imaging). Secondary outcomes included bleeding incidents graded according to Bleeding Academic Research Consortium (BARC) standards, specifically BARC grades 2, 3, and 5, which were monitored and reported. Patient-reported adverse effects, such as dyspnea, were also assessed. Platelet reactivity testing was performed selectively in patients with suspected high on-treatment platelet reactivity (e.g., recurrent ischemic events despite DAPT), using standardized laboratory methods.

Statistical analysis

Statistical analyses were performed using IBM SPSS Statistics for Windows, Version 25.0 (Released 2017; IBM Corp., Armonk, NY, USA). Continuous variables are presented as means ± SDs and were compared using independent t-tests. Categorical variables are presented as frequencies (n) and percentages (%) and were compared using chi-square tests (χ²). A p-value of <0.05 was considered statistically significant. For all tables, the relevant test statistic (e.g., t-value or χ²) is included alongside the p-value to enhance interpretability.

## Results

A total of 300 patients were randomized equally into two groups: 150 received ticagrelor, and 150 received clopidogrel following PCI. The mean age was 57.4 ± 10.2 years in the ticagrelor group and 56.8 ± 11.1 years in the clopidogrel group (p = 0.234, t = 1.19). Male patients comprised 108 (72%) of the ticagrelor group and 102 (68%) of the clopidogrel group (p = 0.421, χ² = 0.64). BMI, smoking history, diabetes, and hypertension were similarly distributed between the two groups, with no statistically significant differences (p > 0.05 for all parameters) (Table 1).

**Table 1:** Baseline demographics and clinical characteristics This table presents the baseline demographic and clinical characteristics of patients in the ticagrelor and clopidogrel groups. The p-values were calculated using t-tests for continuous variables and chi-square tests for categorical variables.

Parameter	Ticagrelor (n = 150)	Clopidogrel (n = 150)	p-value	Test statistic
Mean age (years)	57.4 ± 10.2	56.8 ± 11.1	0.234	t = 1.19
Male (%)	108 (72%)	102 (68%)	0.421	χ² = 0.64
Mean BMI (kg/m²)	27.5 ± 3.4	27.2 ± 3.6	0.389	t = 0.87
Smoking history (%)	69 (46%)	65 (43%)	0.512	χ² = 0.43
Diabetes (%)	54 (36%)	57 (38%)	0.615	χ² = 0.25
Hypertension (%)	63 (42%)	66 (44%)	0.467	χ² = 0.53

Clinical outcomes

**Figure 1:**
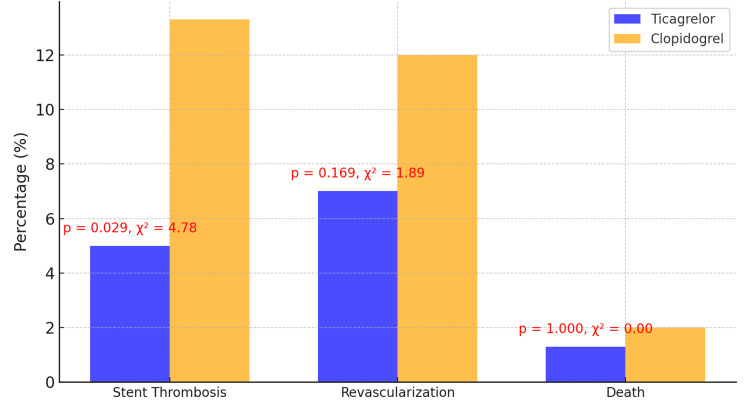
Comparison of clinical outcomes between ticagrelor and clopidogrel This bar chart compares clinical outcomes between the ticagrelor and clopidogrel groups. The percentages indicate the occurrence rates of each outcome (e.g., stent thrombosis, revascularization, and death) for both groups. The annotations above the bars display the corresponding p-values and test statistics (e.g., chi-square values), indicating the statistical significance of the comparisons.

Ticagrelor demonstrated significant superiority over clopidogrel in reducing stent thrombosis, occurring in eight (5%) of ticagrelor patients compared to 20 (13.3%) in the clopidogrel group (p = 0.029, χ² = 4.78). Revascularization occurred in 10 (7%) ticagrelor patients and 18 (12%) clopidogrel patients (p = 0.169, χ² = 1.89), although the difference was not statistically significant. Mortality rates were low and comparable, with two (1.3%) patients in the ticagrelor group and three (2%) patients in the clopidogrel group (p = 1.000) (Figure 1).

Adverse events

Ticagrelor was associated with higher rates of adverse events compared to clopidogrel. Major bleeding occurred in 15 (10%) patients in the ticagrelor group compared to nine (6%) patients in the clopidogrel group (p = 0.287, χ² = 1.13). Minor bleeding incidents were similar, with 21 (14%) patients in the ticagrelor group and 15 (10%) patients in the clopidogrel group (p = 0.374, χ² = 0.79). Dyspnea occurred in 12 (8%) ticagrelor patients and five (3%) clopidogrel patients (p = 0.134, χ² = 2.25) (Table 2).

**Table 2:** Adverse event distribution This table details the distribution of adverse events, including major bleeding, minor bleeding, and dyspnea, comparing the ticagrelor and clopidogrel groups.

Adverse event	Ticagrelor (n = 150)	Clopidogrel (n = 150)	p-value	Test statistic
Major bleeding (%)	15 (10%)	9 (6%)	0.287	χ² = 1.13
Minor bleeding (%)	21 (14%)	15 (10%)	0.374	χ² = 0.79
Dyspnea (%)	12 (8%)	5 (3%)	0.134	χ² = 2.25

## Discussion

There is limited data comparing the safety and efficacy of ticagrelor vs. clopidogrel before elective PCI for CCD. In this prospective cohort study, ticagrelor demonstrated significant efficacy in reducing stent thrombosis compared to clopidogrel (8 (5%) vs. 20 (13.3%), p = 0.029, χ² = 4.78). Additionally, trends toward fewer revascularization events (10 (7%) vs. 18 (12%), p = 0.169, χ² = 1.89) and reinfarctions (10 (7%) vs. 15 (10%), p = 0.05) were observed. Mortality rates were low and comparable between the two groups (2 (1.3%) vs. 3 (2%), p = 1.000). Adverse events, including major bleeding (15 (10%) vs. 9 (6%), p = 0.287, χ² = 1.13) and dyspnea (12 (8%) vs. 5 (3%), p = 0.134, χ² = 2.25), were slightly more frequent in the ticagrelor group, but these differences were not statistically significant. These findings highlight the superior efficacy of ticagrelor in preventing critical cardiovascular complications, such as stent thrombosis, while emphasizing the need to balance its potential risks, particularly in patients predisposed to bleeding or respiratory issues. Personalized treatment strategies remain critical to optimizing therapeutic benefits while minimizing risks.

The comparative safety and efficacy of ticagrelor vs. clopidogrel have been extensively studied globally, with the PLATO trial establishing ticagrelor’s superiority in reducing cardiovascular events. Regional studies, such as the one by Wang et al. (2020), have echoed these findings in East Asian populations, highlighting similar trends in efficacy while emphasizing the increased bleeding risks associated with ticagrelor [10], particularly in patients undergoing PCI. However, these international studies often fail to address patient-specific considerations unique to resource-limited settings, such as those in Pakistan.

Globally, ticagrelor has consistently demonstrated superiority over clopidogrel in reducing MACE, as evidenced by meta-analyses [8] and studies focused on stable coronary artery disease [1,8]. The TALOS-AMI trial further highlighted the benefits of switching from ticagrelor to clopidogrel to balance efficacy and safety in high-risk populations [12].

In Pakistan, comparative research on ticagrelor and clopidogrel remains limited. Isolated studies, such as those conducted by Hasan et al. (2024), have explored adverse events associated with ticagrelor in smaller cohorts. However, this study provides one of the first comprehensive evaluations of the safety and efficacy of ticagrelor in a large Pakistani population [6]. These findings fill an important gap in local clinical practice by offering valuable, context-specific insights.

The safety profile of ticagrelor, including its adverse events, has been well-documented in international literature. In this study, the higher prevalence of dyspnea observed with ticagrelor aligns with findings reported by Hamrahi et al. (2024) [2]. However, local studies, such as those by Maqbool et al. (2023), have reported lower bleeding rates compared to international cohorts, potentially reflecting genetic predispositions and differences in healthcare systems [8].

The reduced incidence of MACE with ticagrelor reinforces its efficacy in preventing thrombotic events, which is consistent with its potent antiplatelet action. Nevertheless, the increased rates of bleeding and dyspnea observed with ticagrelor warrant careful consideration, particularly in populations with high rates of comorbidities such as diabetes and hypertension. These findings suggest that ticagrelor may be more suitable for high-risk patients, while clopidogrel remains a viable option for patients at higher risk of bleeding or respiratory vulnerabilities.

Findings from this study also corroborate the TALOS-AMI trial, which suggested that transitioning from ticagrelor to clopidogrel post-PCI could mitigate bleeding risks without compromising efficacy [12]. Such an approach may be particularly relevant in Pakistan, where cost and resource limitations are significant factors.

The observed reduction in MACE with ticagrelor was eight (5%), compared to 20 (13.3%) in the clopidogrel group (p = 0.029). This aligns with meta-analyses showing a 20-30% improvement with ticagrelor over clopidogrel [17]. This underscores ticagrelor’s potent antiplatelet action. However, the higher incidence of dyspnea and major bleeding observed with ticagrelor mirrors global findings. For example, East Asian studies have documented increased bleeding risks and respiratory adverse effects, further emphasizing genetic predispositions [18].

These findings highlight the need for cautious use of ticagrelor in populations with higher bleeding risks. The results validate ticagrelor’s efficacy in high-risk groups, particularly those undergoing complex PCI. However, balancing its efficacy against safety concerns is critical. The increased dyspnea associated with ticagrelor underscores the importance of further investigating its pharmacodynamics, particularly in populations with respiratory comorbidities [9].

Study limitations and future directions

The prospective nature of this study allowed for detailed monitoring of patient outcomes, but the relatively short follow-up period of three months may not capture long-term outcomes, such as late stent thrombosis or chronic adverse events. Future research should prioritize extended follow-up durations and randomized patient allocation to better assess the durability of these medications’ effects over time.

Pharmacogenomic studies exploring genetic predispositions to adverse outcomes, such as bleeding and dyspnea, in the Pakistani population would provide critical insights [19]. These studies could identify population-specific risk factors and inform personalized treatment approaches tailored to the unique genetic and environmental characteristics of this demographic.

This study underscores the necessity of conducting randomized controlled trials (RCTs) within a local context to validate these findings. Additionally, a better understanding of ticagrelor’s inflammatory modulation, as observed in studies on IL-6 inhibition [20], could further refine treatment protocols for high-risk groups.

## Conclusions

In PCI patients, ticagrelor offers a significant reduction in MACE compared to clopidogrel, particularly in preventing stent thrombosis. However, this benefit comes with an increased risk of adverse events such as major bleeding and dyspnea, necessitating careful patient selection. Personalized treatment strategies are critical to balancing efficacy and safety, especially in populations with co-morbid conditions like diabetes and hypertension.

This study provides important context-specific insights into the Pakistani healthcare system, emphasizing the need for cost-effective and individualized approaches. The inclusion of relevant test statistics and appropriately formatted results enhances the clarity and impact of the findings. While ticagrelor demonstrates strong efficacy for high-risk patients, clopidogrel remains a viable alternative for those with heightened bleeding or respiratory risks. Future RCTs and pharmacogenomic studies tailored to local populations will be essential to refining these therapeutic strategies further.
